# Computing Arm Movements with a Monkey Brainet

**DOI:** 10.1038/srep10767

**Published:** 2015-07-09

**Authors:** Arjun Ramakrishnan, Peter J. Ifft, Miguel Pais-Vieira, Yoon Woo Byun, Katie Z. Zhuang, Mikhail A. Lebedev, Miguel A.L. Nicolelis

**Affiliations:** 1 Department of Neurobiology, Duke University, Durham, NC, USA; 2 Duke University Center for Neuroengineering, Duke University, Durham, NC, USA; 3 Department of Biomedical Engineering, Duke University, Durham, NC, USA; 4 Department of Psychology and Neuroscience, Duke University, Durham, NC, USA; 5 Edmund and Lily Safra International Institute of Neurosciences of Natal, Natal, Brazil

## Abstract

Traditionally, brain-machine interfaces (BMIs) extract motor commands from a single brain to control the movements of artificial devices. Here, we introduce a Brainet that utilizes very-large-scale brain activity (VLSBA) from two (B2) or three (B3) nonhuman primates to engage in a common motor behaviour. A B2 generated 2D movements of an avatar arm where each monkey contributed equally to X and Y coordinates; or one monkey fully controlled the X-coordinate and the other controlled the Y-coordinate. A B3 produced arm movements in 3D space, while each monkey generated movements in 2D subspaces (X-Y, Y-Z, or X-Z). With long-term training we observed increased coordination of behavior, increased correlations in neuronal activity between different brains, and modifications to neuronal representation of the motor plan. Overall, performance of the Brainet improved owing to collective monkey behaviour. These results suggest that primate brains can be integrated into a Brainet, which self-adapts to achieve a common motor goal.

BMIs are computational systems that link biological circuits to external devices, such as computer cursors, robotic prostheses and communication aids^1^. Heretofore, BMIs have been utilized either: (i) to extract motor signals from neural activity and convert them to the movements of external devices^2^,^3^,^4^,^5^, (ii) to deliver sensory signals from the environment to the brain^6^,^7^,^8^, or (iii) to combine both operations and enable bidirectional communications between the brain and machine^9^. In each of these implementations, a BMI serves as an accessory to a single brain. Recently an entirely new direction was proposed for BMI research – a brain to brain interface (BtBI)^10^. BtBI allows animal brains to exchange and share sensory and motor information to achieve a behavioural goal^11^,^12^,^13^,^14^,^15^,^16^,^17^. BtBI is a *hybrid* computational system since it incorporates both biological components (the primate brains) and digital parts (the BMI system).

In the present study, we have designed and tested a more elaborate computational architecture which we refer to as a Brainet^10^. Our Brainets involved groups formed by 2-3 monkeys in a shared BMI that enacted conjoint motor behaviours. Previously, human psychophysics studies have shown that two or more individuals who are performing movements simultaneously often entrain to each other’s behavior, even if they are not explicitly instructed to do so^18^,^19^,^20^,^21^,^22^. However, the neurophysiological mechanisms of such joint actions are not well understood. In particular, we were interested in investigating the possibility that neuronal ensemble could directly control conjoint behaviors enabled by multiple interconnected BMIs.

Our study adds to previous attempts to overcome limitations of one individual confronted with a high processing load by mixing contributions of multiple individuals^23^,^24^,^25^,^26^,^27^,^28^,^29^. Particularly relevant to our present work, several EEG studies^13^,^30^,^31^,^32^,^33^,^34^ have combined brain derived signals from multiple subjects to enhance visual discrimination, motor performance, and decision making. A recent EEG study^30^ has implemented shared control that involved dynamic collaboration of multiple individuals in real time to achieve a common goal. However, in none of these EEG experiments participants interacted with each other over a long term. Moreover, no large-scale intracranial cortical recordings were obtained in order to investigate the neurophysiological mechanism underlying conjoint motor behaviour. As such, the present study is the first to implement Brainets based on the chronic extracellular recordings of up to 783 individual cortical neurons distributed across multiple rhesus monkey brains.

To this end, we have implemented Brainet architectures consisting of two (B2) (Fig. 1B,C) or three (B3) (Fig. 1A,D) monkey brains. These Brainets (i) controlled 2D/3D movements of an avatar arm by sharing signals derived from multiple brains (Fig. 1B,D), (ii) partitioned control by delegating subtasks to different subjects (Fig. 1C,D) and (iii) enabled a super-task that was composed of individual BMI tasks (Fig. 1A,D).

## Results

**Figure 1:**
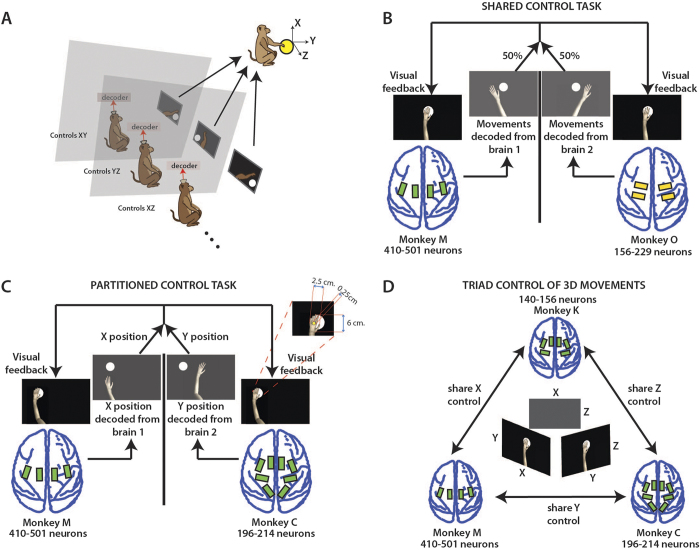
Experimental setups for B2 and B3 experiments. (**A**) Monkeys were seated in separate rooms, each facing a computer monitor showing the virtual avatar arm (inset in C) from a 1st person perspective. (**B**) Shows the shared control task, (X,Y) position of the virtual arm was decoded during centre-out movements from the two monkeys’ brains with each given 50% control of the arm. Electrode array location shown on brains. (**C**) Shows the partitioned control task. X position of the arm was decoded from one monkey and Y position from the other during centre-out movements toward targets. (**D**) Shows the 3-monkey task. Each monkey observed and had 50% control over 2 of the 3 dimensions (X, Y, or Z). Together, the three monkeys must accurately perform a 3-D centre-out movement to achieve reward. Drawings by Miguel A.L. Nicolelis.

Four monkeys participated in the experiments. They were chronically implanted with multielectrode arrays in motor (M1) and somatosensory cortices (S1)^1^,^35^. Extracellular electrical activity from 570-783 neurons was sampled chronically from monkeys M (410-501 neurons), C (196-214), O (156-229), and K (140-156) up to five years after implantation^36^. B2s operated using at least 550 neurons, while the B3s operated with at least 775 neurons. During experimental sessions, monkeys sat in separate rooms where each of them viewed a realistic monkey avatar arm on a computer screen (Fig. 1A). Movements of the avatar arm were generated using either a hand-held joystick (hand control) or from modulations in VLSBA in a mode called brain control^4^,^5^. In brain control, a neural decoding algorithm called the unscented Kalman filter (UKF)^37^ extracted reach kinematics from neuronal ensemble activity in real-time.

### Experiment 1: Shared control

In the first experiment (shared control task, Fig. 1B), two monkeys worked together to move the avatar arm in 2D space. Each monkey contributed equally (50%) to both the X and Y coordinates. The monkeys moved the avatar arm from the screen centre towards a circular target (Movies S1, S2). These movements were enacted either using the joystick (dyad M&O) or the Brainet (monkey dyads M&O and M&C). To obtain training data for brain control, we started each session with a passive observation epoch during which monkeys watched the computer-generated avatar arm movements along centre-out trajectories^38^. One UKF was fit per monkey, and the UKF outputs (X and Y coordinates) were combined across monkeys to produce the conjoint brain-controlled movements of the avatar arm.

**Figure 2:**
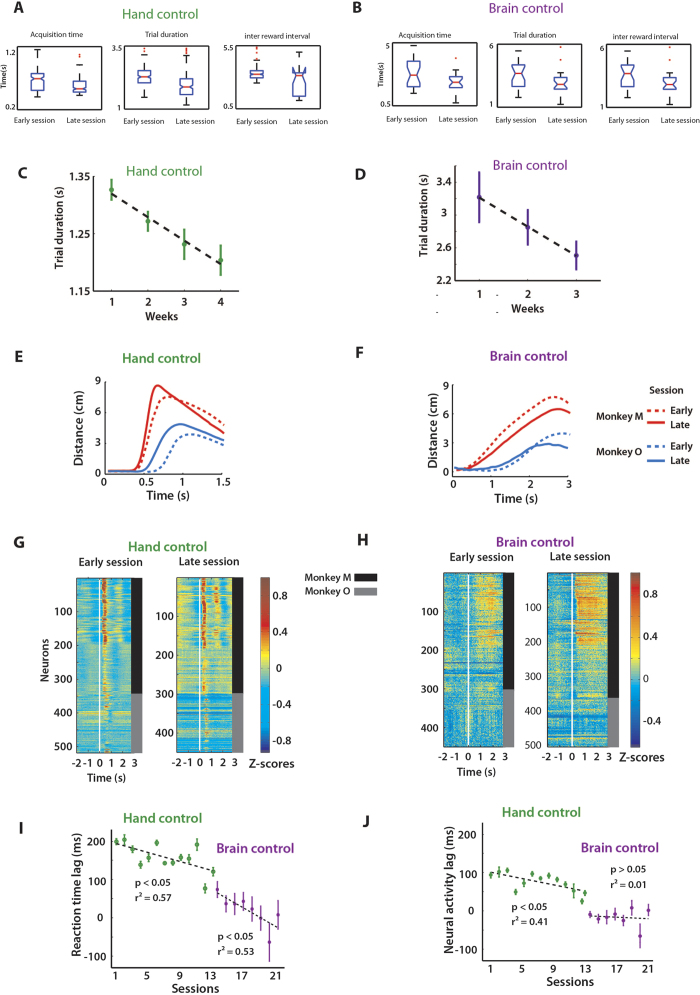
B2 shared control task. (**A**,**B**) Boxplots comparing target acquisition time (left panel), trial duration (centre panel), inter-reward interval (right panel) in hand control (**A**) and in brain control (**B**). (**C**,**D**) Changes in trial duration with conjoint training across weeks. Mean ± SEM trial duration for each of the four weeks of hand control experiments (**C**) and three weeks of brain control (**D**) for dyad M&O. Dashed line shows linear regression fit. Trial duration reduced significantly with improvement in coordination between monkeys. (**E**,**F**) Trial-averaged movement profiles from an early session (dashed) and a late session (solid) for monkey M (red) and O (blue) aligned to the time of target onset (time = 0 ms.). Plots are shown up to 1.5 s for hand control (with mean target acquisition time of 0.8 s) and 3 s for brain control (with mean target acquisition time of 2.2 s). The target was located 9 cm from the centre (y-axis). As the dyad trained together over a span of 7 weeks the reaction time lag, the time of movement onset of monkey O relative to monkey M, decreased during both hand control (**E**) and brain control (**F**) (**G**,**H**) Neural activity PETH from an early session and a late session for hand control (**G**) and brain control (**H**) Each row is a single neuron, colour denotes normalized firing amplitude. Monkey M and O neurons marked by black (**M**) and grey (**O**) vertical bands. Notice that neuronal modulations are more intense and synchronized across the monkey pair in the later session as compared to the early session. **(I**) Changes in reaction time lag between monkey M and O across experiments. Lag was derived from peak of cross-correlation of two monkeys’ behavioural traces (**E**,**F**) Trends fit with linear regression. As the dyad trained together the reaction time lag reduced to 10 ± 27 ms. (**J**) Lag in neural activity between two monkeys over the same experiments as in (**E**) computed again by finding the peak of cross correlogram on each session. As the dyad trained together the reaction time lag reduced to 4.2 ± 5 ms.

The behaviour of the dyad MO improved significantly with training (Fig. 2A–D). Over several weeks of training (4 weeks of hand control followed by 3 weeks of brain control), we observed a significant reduction in target acquisition time between early and late sessions (Figs. 2A,B) (p < 0.001 and p < 0.01; KS test for hand control and brain control, respectively), trial duration (p < 0.001 and p < 0.02) and inter-reward interval (p < 0.004 and p < 0.02). The mean trial duration across weeks of training significantly decreased for both hand control (∆t = 0.13 s, 1-way ANOVA: p < 0.05) (Fig. 2C) and brain control training (∆t = 0.7 s, 1-way ANOVA: p < 0.05) (Fig. 2D). To assess the performance of the B2 in manual control and brain control modes, we also calculated performance metrics reported in the previous literature^39^,^40^. Table 1 shows the overall mean target acquisition time, target hit rate and the movement precision required, which was quantified as the ratio of target size to the size of the workspace^40^.

As monkeys M and O learned the shared control task, their coordination improved in both hand control and brain control modes (Fig. 2E–J). In the initial session, with monkey M initiating movements earlier than monkey O (Fig. 2E,I; hand control), the lag between the monkeys’ reaction times was 200 ± 12 ms (mean ± SEM). With conjoint training, reaction time lag between the two monkeys steadily decreased to 10 ± 27 ms over 21 sessions (13 sessions of hand control and 8 sessions of brain control, Fig. 2I). This gradual reduction in lag was statistically significant (hand control: linear regression: r^2^ = 0.57; p < 0.01, brain control: linear regression: r^2^ = 0.53; P < 0.05). As the monkey dyad became more coordinated, the cortical neural activity lag between the two brains decreased and stabilized near zero (Fig. 2G,H,J). More specifically, cortical activity lag decreased during hand control (linear regression: r^2^ = 0.41; P < 0.05) and remained approximately zero (4.2 ± 5.1 ms) throughout brain control (purple data in Fig. 2J, linear regression: R^2^ = 0.01; P > 0.05).

For the other dyad (M&C), the behavioural responses were synchronous and lag remained close to zero (3 ± 35 ms; linear regression: P = 0.44) throughout all sessions (8 sessions) (Fig. S1, Movie S2). Dyad M&C performed brain control after passive observations of the avatar arm movements and without a requirement to coordinate their behaviours in the manual task. This training sequence probably aided better coordination between the subjects. Table 1 shows the mean target acquisition time, target hit rate and the movement precision required for the dyad M&C.

**Figure 3:**
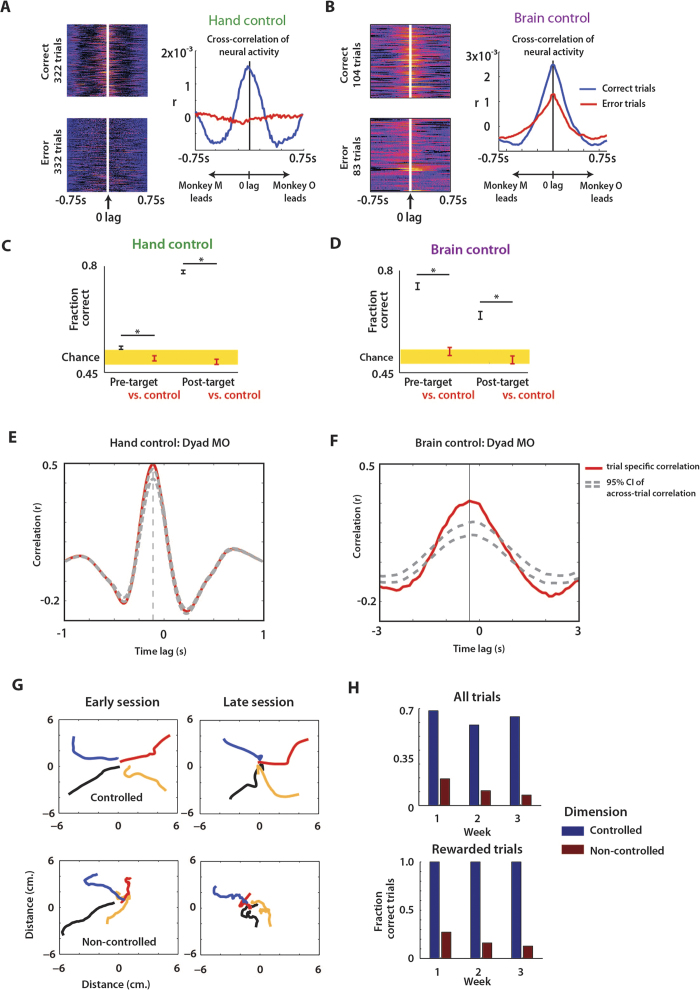
(A-B) Cross correlogram of Monkey M vs. Monkey O neural activity on correct trials (upper left panel) and error trials (lower left panel) for both a representative hand control (**A**) and brain control session (**B**) Mean cross correlogram for the correct and error trial group shown in panel on right of each panel. The correlation is higher in correct trials indicative of increased synchronous neuronal modulations between monkeys. (**C**,**D**) k-NN prediction of trial outcome (reward or error) using the mean neuronal cross correlogram on a single trial (i.e. left panels in A-B) between the two monkeys either prior to target appearance (“Pre-target”) or after target appearance (“Post-target”). Chance level prediction (95% confidence interval) shown in yellow band. * denotes P < 0.01, unpaired t-test. Neuronal synchrony between monkeys before and after target onset was predictive of trial outcome. (**E**,**F**) Extra correlation analysis. Velocity profile in a trial for dyad M&O were cross-correlated. The average cross correlation (trial specific correlation, red trace) was estimated for all trials: hand control (**E**) and brain control (**F**) Extra correlation was the excess correlation (in the red trace) that cannot be accounted for by the distribution of across-trial correlation (enclosed by the grey dashed lines). The vertical line shows the time lag at peak correlation. (G-H) Partitioned control task: (**G**) Panels show the average trajectory to the 4 target locations, denoted by colour, from the first (left column) and the last (right column) session. Trajectories derived from the monkeys’ controlled axes predictions (controlled) are shown in the upper panels and non-controlled axes predictions (non-controlled) are shown in the lower panels. Non-controlled traces were initially more similar to the controlled traces (left panels) but over time became shrunk and convoluted (right panels). (**H**) The fraction of rewarded trials was computed based on trials when the avatar arm is moving according to the predictions of the controlled axis (blue) or the non-controlled axes (red). Mean fraction correct trials shown for each of the three weeks of experiments. Shown separately is fraction correct amongst all trials (upper panel) and only among trials where a reward was achieved (lower panel). The percentage of trials in which the complementary trace reached the target (red bars) decreased significantly over training.

Concurrent changes in neural activity during Brainet operation could be related to multiple factors: common visual feedback of the avatar arm movements, common representation of the target (spatial location and temporal onset), and common BMI outputs (movements to the same location). To understand if the interplay between the common sensory input and common motor behaviour could account for the genesis of concurrent neural activity we carried out further analyses. First, we investigated the relationship between the behavioural outcomes in each trial and concurrent neuronal modulations between the monkeys in the same trials. We found correct task outcomes were associated with greater neural correlations between monkeys, both in hand control (Fig. 3A; P < 0.01; unpaired t-test) and brain control (Fig. 3B; P < 0.01). To clarify the role of concurrent neuronal activity further, we conducted an analysis where a k-NN classifier predicted the trial outcome, based on the neural correlations between monkeys, both before (700 ms before target appearance until target appearance) and after target onset (from 100 to 800 ms after target appearance). The neuronal correlations were significant predictors of trial outcome (P < 0.05, 1-proportion z-test) not only after target appearance but also before (Fig. 3C,D). Notably, the effect of neural synchrony on the trial outcome was stronger in brain control mode (Fig. 3D). We suggest that the presence of neural correlation prior to target onset indicated that the monkeys attended to the avatar arm since the common visual input from the avatar arm was the only source of neural correlation during that period. This interpretation in terms of an increased attention agrees well with the better performance on the trials with high neural correlation: the more the monkeys attend to the avatar arm and the other screen events, the better they perform.

Next, we examined whether the increase in concurrent neuronal activity between the two monkeys could emerge due to the fact that the two animals move simultaneously, without necessarily paying attention to each other, following visual stimulus onset. If this scenario were true, there would not be any trial specific neuronal correlation beyond the level that could be observed if the behavioural trials of one of the monkeys were randomly shuffled (provided that target locations still matched for two monkeys). To investigate this possibility, we used movement velocities as a proxy to test the correlation between monkeys. To determine the additional component to the coordination between the monkeys, over and above the coordination that comes about as a result of jointly performing reaching movements to a specific target location, we calculated a parameter called “extra-correlation”. Extra-correlation is the correlation between the two monkeys’ movement velocities in a trial that cannot be explained by the across-trial correlation. Across-trial correlation was computed by correlating velocity of movement of a monkey from one trial with that of the partner monkey on another randomly chosen trial in which the monkeys moved to the same target location. This was performed for several such combinations of trials (40-50 combinations) to obtain a distribution of the across-trial correlation between the velocities of the dyad. We observed that extra correlation in movement velocities, which was significant in 3/13 sessions during hand control (Fig. 3E), was however, significant during all brain control sessions for both dyad MO (Fig. 3F) and MC (Fig. S2). These results suggest that increases in concurrent activity is not only due to motor outputs triggered by the same visual stimulus, but also due to a significant trial specific coordination in movement velocities, especially during brain control.

### Experiment 2: Partitioned control

In the next experiment, a B2 (dyad M&C) performed a partitioned control task where each monkey performed a subtask of a 2D movement (Fig. 1C). One monkey controlled only the X position of the avatar arm (X-monkey) and the other monkey controlled only the Y position (Y-monkey). The targets appeared on the diagonals at 45°, 135°, 225°, or 315°, which meant that both the X-monkey and Y-monkey had to simultaneously and accurately enact movements along their individual axes to achieve a correct trial. Each session started with a period of passive observations of the avatar arm movements. Based on the data recorded during this period, one UKF was fit per monkey. During brain control, the UKF outputs (X for monkey M, and Y for monkey C) were combined. The unused outputs (Y for monkey M, and X for monkey C) were computed, but not shown to the monkeys as any kind of feedback. We compared the used and unused X and Y outputs in an offline analysis to estimate cortical adaptation to preferentially represent the coordinate being controlled through the BMI.

During brain control mode, despite being given control of only one axis, a monkey could in principle generate motor plans at the neural level that encoded movements in both the X and Y dimensions. Alternatively, each monkey could parse the 2D trajectory into a controlled dimension (which it had to attend to) and non-controlled dimension (which it could disregard). For example, if monkey M controlled movements along X-axis, we asked how this animal’s cortical neurons changed their contribution to movements along Y-axis, which they did not control. That provided us with a way to quantify how much functional plasticity was occurring in the monkeys’ cortex as a result of partitioned Brainet operation. To measure that, we first plotted the actual avatar movement traces generated by the B2 (Fig. 3G; upper panels) which corresponds to the axes the monkeys controlled (X for monkey M, and Y for monkey C). We then compared this plot with the traces for the complementary pair of dimensions (Y for monkey M, and X for monkey C; Fig. 3G: lower panels). We found that the members of the B2 contributed more along the dimension they controlled and less along the dimension they did not control. This result is evident from the comparison of the actual avatar movement traces generated by the B2 (Fig. 3G; upper panels) to the complementary traces (Fig. 3G; lower panels). These traces were similar in the early sessions (Fig. 3G; left panels) but diverged over time (Fig. 3G, right panels). By the 3^rd^ week of training, average complementary traces were shrunk and convoluted compared to the average actual traces. As a result of this cortical adaptation, the complementary trajectories seldom reached the target. The percentage of trials in which the complementary trace reached the target (red bars in Fig. 3H) decreased from 26% to 12% over training (regression, r^2^ = 0.42 upper panel of Fig. 3H, P < 0.05). Similar reduction was observed when only the rewarded trials were included (r^2^ = 0.38, P < 0.05, lower panel Fig. 3H).

### Experiment 3: Brainet triad control of an avatar arm in 3-D space

In the third experiment, we built a 3D super-task out of individual 2D BMI tasks. Each of the three monkeys forming a B3 viewed the 2D projection of a spherical target in 3D space, from an X-Y, Y-Z, or X-Z reference frame (Fig. 1D). The monkeys moved the avatar arms on their displays in their 2D space to the projected target location. As they reached the projected target, a 3D avatar arm that received inputs from all the three UKF decoders reached the true target location in 3D space (Movie S3). This design reduced task difficulty for any one individual monkey by breaking down the 3D task into simpler 2D subtasks.

Each session started with a period of passive observation during which each monkey watched the computer-generated avatar arm perform the corresponding subtask. A UKF model was fit per monkey and its subtask, and the individual monkey outputs (X, Y for monkey M; Y, Z for monkey C; and X, Z for monkey K) were combined to produce 3D movement of the avatar arm. Put another way, during brain control, pairs of monkeys shared equal control over one of the axes: Monkeys M and K controlled the X-axis, monkeys M and C controlled the Y-axis, and monkeys C and K controlled the Z-axis.

**Figure 4:**
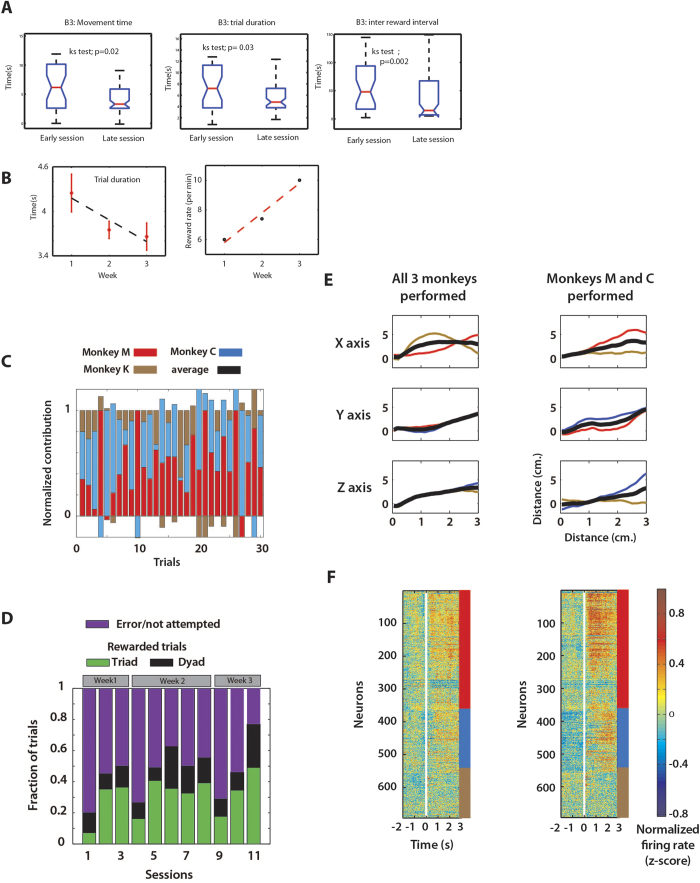
Triad (B3) control of 3D movements. (**A**) Boxplots comparing target acquisition time (left panel), trial duration (centre panel), inter-reward interval (right panel). (**B**) Reduction in trial duration (left panel) and concurrent increase in the reward rate (right panel) with conjoint training across weeks. (**C**) Normalized contribution of each of the three monkeys across a representative subset of 30 trials. The relative contribution of each monkey varied from trial to trial. (**D**) Fraction of trials that were correctly performed by a dyad (black) as a triad (green), or incorrectly (purple) shifted across the 11 triad experiments. The fraction of total trials with a rewarded outcome in which all three monkeys contributed (green), or those in which two monkeys contributed (black) increased significantly within each week and across sessions whereas the fraction of erroneous/unattempted trials reduced significantly. (**E**,**F**) Decoded trajectories and neural data from the triad experiment. (**E**) Mean X,Y,Z traces produced by individual monkeys shown separately by colour among trials where all monkeys contributed (left column) or when only monkeys M and C contributed (right column). Mean X,Y,Z (the value used to move avatar) shown in black. Distance to target in each axis is 5 screen-cm. When one monkey (monkey K) opted out, the working dyad generated higher-amplitude trajectories (Right column, X axis and Z axis) as opposed to when all the members contributed (left column). (**F**) PETHs aligned on target onset for same trial subsets as in (**E**) Rows represent individual neurons and colour indicates normalized firing rate (z-score). Neurons from different monkeys marked by colour along right edge (same colours as in (**E**)). Increased effort by the working dyad also resulted in stronger cortical modulations between the members (right panel) as compared to when all the members contributed (left panel).

Clear behavioural improvements occurred during the brain control epochs over a span of three weeks of training. We observed a significant reduction in target acquisition time (p < 0.02; KS test), trial duration (p < 0.03; KS test) and inter-reward interval (p < 0.002; KS test) between early and late sessions (Fig. 4A). The mean trial duration significantly decreased (4.25 to 3.65 s, 1-way ANOVA: p < 0.05, Fig. 4B panel on the left) over the span of three weeks and the mean reward rate increased from 6 to 10 trials per minute (Fig. 4B, panel on the right). Across 11 sessions, the B3 significantly improved its performance from 20% correct trials in the first session to 78% correct trials in the last session (P < 0.01, 1-way ANOVA with bootstrapping, green+black bars in Fig. 4D).

Table 1 shows the overall mean target acquisition time, target hit rate and the movement precision required for the B3. Due to the addition of the third dimension, movement precision required of B3 was scaled by a factor of 5.5 as compared to B2. However, the target acquisition times increased only by a factor of 1.9 (Dyad MC vs. Triad MCK). Target hit rate remained nearly the same for both paradigms. The increase in target acquisition times indicates the cost of synchronizing an additional monkey into a B3. However, as a result of the design, the B3 performed reach movements in conditions that required greater movement precision, without compromising the hit rate.

Even though the relative contribution of each monkey varied from trial to trial (Fig. 4C), the highest rate of success was attained when all three monkeys contributed (Fig. 4D). Furthermore, the percent of total trials with a rewarded outcome in which all three monkeys contributed to the final movement outcome (green bars in Fig. 3D) grew from 7% to 50%, the largest increase of any possible dyad or triad tested in this study. The increase was significant within each week of training (post-hoc Tukey test: P < 0.05) and across weeks (1-way ANOVA with bootstrapping: P < 0.001).

The B3 design was resilient to any one individual monkey underperforming in a given trial because any monkey dyad could successfully move the avatar arm to the true target location in 3D space. We observed that the number of rewarded trials for the dyad M&C was lower as compared to the triad but improved with sessions as well (from 12% to 27%, P < 0.01, 1-way ANOVA with bootstrapping). This improvement suggests that the B3 could benefit from the redundancy that was built into the design in the form of shared control. However, when one monkey opted out, the working dyad would need to produce higher-amplitude trajectories (Fig. 4E, right column) as opposed to when all the members contributed (Fig. 4E, left column). Increased effort by the working dyad also resulted in stronger cortical modulations between the members (Fig. 4F, right panel) as compared to when all the members contributed (Fig. 4F, left panel).

### Neuron-dropping curves: decoding improves with number of neurons

**Figure 5:**
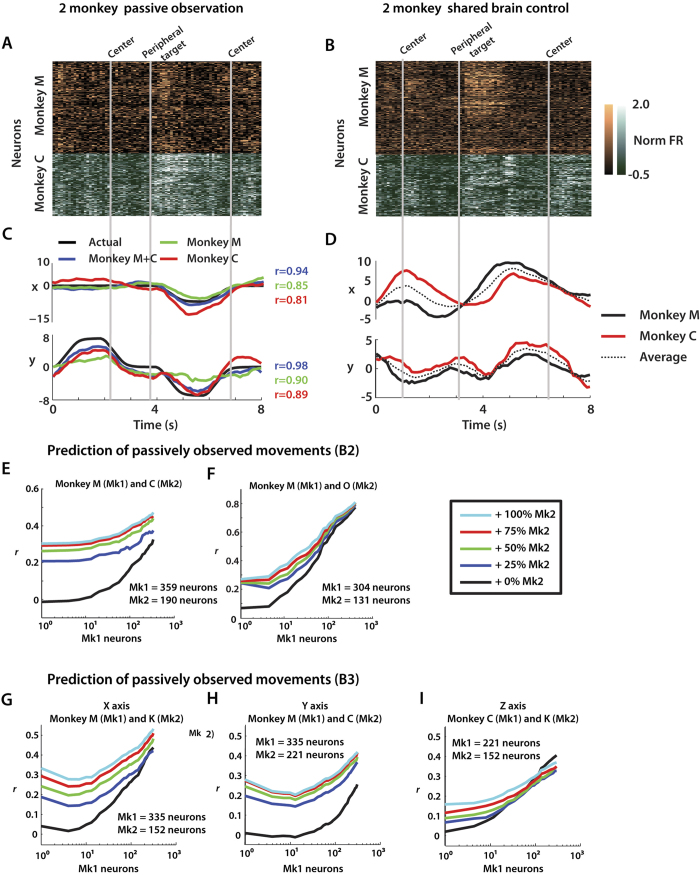
Neuronal representations during B2 and B3 shared control experiments. Neuronal modulations from monkey M (orange) and C (green) during a 10 second window of passive observation. (**A**) and brain control (**B**) Centre and peripheral target onset times are denoted by grey vertical lines. Cortical ensembles in each monkey exhibited clear task-related activity during both passive observation and brain control modes. (**C**) Passively observed trajectory (black) compared with predicted trajectory using only Monkey M (green), only Monkey C (red), or both (blue) neuronal ensembles. Grey vertical lines from (**A**) again denote relevant task events. Accuracy of arm movement decoding improved when VLSBA was recorded and combined from multiple brains. (**D**) Decoded X and Y trajectories from monkey M (black, solid) and monkey C (red), as well as the average of the two (dotted) during 8 second window of brain control experiment. (**E**,**F**) Neuron dropping curves (NDC) showing effect of ensemble size of each of the two monkeys on prediction accuracy during B2 passive observation. The number of neurons used from Mk1 marked by x-axis. The percent of Mk2 population used for predictions denoted by colour (see Legend). Accuracy of predictions measured as correlation coefficient *r*. The decoding accuracy benefited from mixing the contributions from different brains as well as the overall neuronal mass. (**G**-**I**) Same NDC analysis as (**E**,**F**) except for prediction of X, Y, and Z position during B3 passive observation. Prediction of X (G), Y (H), and Z (I) shown separately. Again (as seen in E-F), the decoding accuracy benefited from mixing the contributions from different brains as well as the overall neuronal mass.

During B2 and B3 operations, cortical ensembles in each monkey exhibited clear task-related activity during both passive observation (Fig. 5A,C) and brain control modes (Fig. 5B,D). Analysis of these neural signals confirmed that the accuracy of arm movement decoding would improve when VLSBA was recorded and combined from multiple brains (Fig. 5C). This finding extends our previous results, using neuron dropping curve (NDC) analyses, where we showed that decoding accuracy consistently improved when larger neuronal samples were recorded from a single brain^4^,^5^. Here, this analysis has been extended to visualize the effect of different relative quantities of two and three brains performing together as part of a Brainet. NDCs were constructed for the decoding of avatar arm position during passive observations and brain control mode for the B2 (Fig. 5E,F) and B3 (Fig. 5G-I). NDCs were plotted as families of curves, where each curve represented decoding accuracy for a neuronal sample composed of a variable-size ensemble from one monkey (Mk1) and a fraction of the full ensemble from the other monkey (Mk2). The NDCs indicated that decoding accuracy benefited from mixing the contributions from different brains as well as the overall neuronal mass. The best accuracy was typically achieved when all neurons from all monkeys were combined (Fig. 5E-I), with the only exception being the Z-axis prediction in B3 (Fig. 5I). When only a small number of Mk1 cells (fewer than 10) were added to Mk2 ensembles during passive observation, decoding accuracy stayed the same or slightly decreased (Fig. 5E-I). However, by scaling to 10^2^ or 10^3^ neurons, this trend shifted such that additional Mk1 neurons added to Mk2 ensembles yielded more accurate predictions.

The NDC analysis indicated that Brainet decoding accuracy improved with an increase in the number of neurons. However, a larger total number of neurons was not the sole factor that contributed to the improvement, particularly, the improvement in performance over time. The total number of recorded neurons per monkey did not change considerably throughout the study (Monkey M: 410-501; Monkey C: 196-214; Monkey O: 156-229; Monkey K: 140-156). Yet, we observed a significant improvement in performance (Fig. 2A,B, Fig. 4A,B), as well as improvement in coordinated behaviour (Fig. 2I) and occurrence of concurrent neuronal activity (Fig. 2J). Coordinated behavior was observed even during hand control when the recorded neurons did not influence behavior.

**Figure 6:**
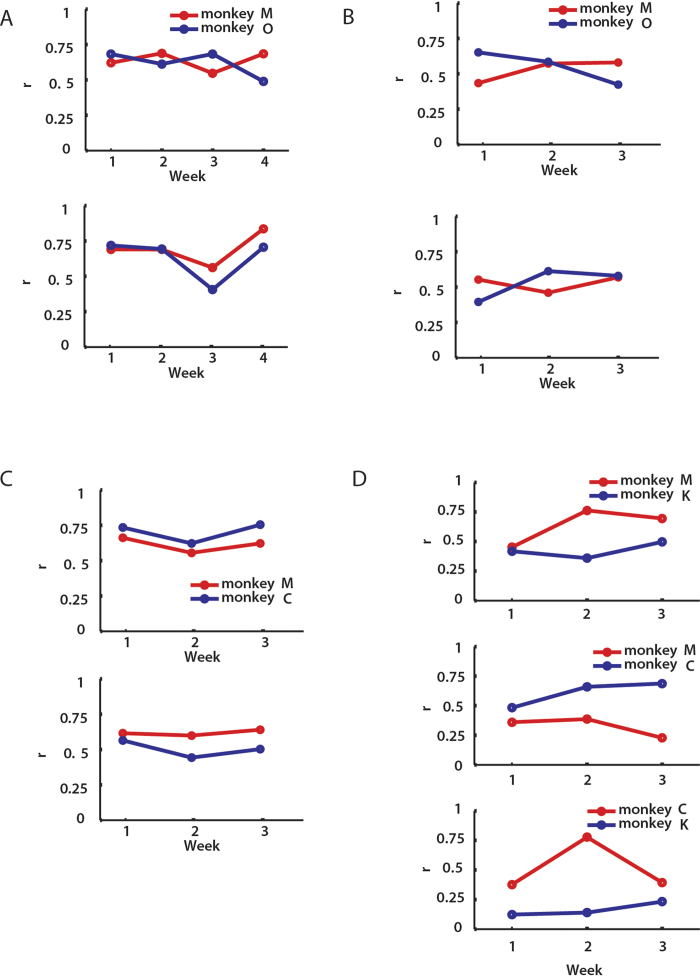
Decoding accuracy in individual monkeys. A UKF decoder trained post hoc predicted monkey’s own movements in every session. (**A**-**C**) Upper panels show the performance of the decoder for the x-component of the trajectories and lower panels for y-component of the trajectories. Each data point represents the average performance for the week. Correlation coefficient *r* was utilized to measure the accuracy of predictions. The performance of the decoder was compared across weeks to monitor the changes in the contribution of the neuronal population during the course of training in the shared control task (**A**,**B**): manual control in (**A**) and passive observation epoch preceding brain control in (**B**) and the partitioned control task (**C**). (**D**) shows the decoder performance for the x-, y- and z- components of the trajectories (top, middle and bottom panels, respectively) in the triad task. Overall, the performance of the decoder remained stable and no consistent trends were observed.

Additionally, improvements in Brainet performance were related to conjoint behavior of the monkeys (as in team work) rather than improvements in individual skills or individual decoding accuracy. All monkeys involved in these experiments had been previously overtrained on the single-BMI task, which makes individual improvements in BMI control an unlikely explanation for the overall improvement of the Brainet performance. Furthermore, we assessed the decoding accuracy in individual monkeys across sessions of manual control (Fig. 6A) and passive observations (Fig. 6B–D). The decoding accuracy showed some fluctuations, which is typical as the motivation levels vary between days of the week. However, there was no systematic trend overall, which contrasts to a steady improvement in Brainet performance over time. These observations suggest that coordinated behavior rather than individual improvements the major factor, which led to better performance.

## Discussion

We have successfully developed a shared monkey BMI that utilized chronic, simultaneous VLSBA recordings from pairs or triplets of primate brains to enact conjoint 2D and 3D movements of a monkey avatar arm. This shared BMI, or Brainet, has scaled the conventional definition of BMI from a technology that derives neuronal signals from a single brain to one that can functionally link multiple brains to coordinate movements in space and time in an unsupervised way.

Previously, joint motor behaviour was studied extensively in human psychophysics studies 18,19,41. Two individuals who are performing movements can become entrained when they mutually affect each other’s behaviour^42^, even if they are not explicitly instructed to do so^22^. In our study, we have shown evidence for both behavioural and neurophysiological entrainment in nonhuman primates that are interacting, indirectly, via a common avatar limb in a virtual environment. The avatar arm provided the same individual action opportunity for all participants involved – also known as simultaneous affordance^21^,^43^. Optimal performance has been proposed to occur in conjoint action when participants can monitor others’ movements^20^ which was facilitated in our experiment through common visual feedback.

The various Brainet configurations tested in this study revealed different types of behavioural and neurophysiological adaptations. Individual monkeys in a B2 could optimize the components of the arm movement they controlled directly over the non-controlled components. At the same time, the dyad adapted spatial components (Fig. 3A,B) while maintaining temporal behaviour/brain coordination. These results suggest that neurophysiological adaptations may be context-dependent.

Shared BMI control introduced redundancy and computational complexity into the Brainet’s operation by allowing the final motor goal to be achieved despite occasional suboptimal behaviour by an individual monkey. Furthermore, as observed in some recent EEG studies^33^,^34^, the NDCs have demonstrated that merging neuronal populations from two/three brains are optimal from the perspective of decoding accuracy.

Another important aspect of our Brainet design is the ability to compensate for performance fluctuations in individual members. For example, in our B3 experiments, we often noticed that monkey C compensated for monkey K’s lack of contribution to movements along the y-axis (Fig. 5D). These observations highlight the potential of a Brainet in assisting individual members to overcome their limitations with the help of the partners.

In conclusion, this study has successfully established a general paradigm for establishing a closed loop shared BMI, formed by two or more brains, through visual feedback. Shared BMI allowed multiple monkey brains to adapt in an unsupervised manner. Based on these evidences, we propose that primate brains can be integrated into a self-adapting computation architecture capable of achieving a common behavioural goal.

## Methods

### Subjects and implants

All studies were conducted with approved protocols from Duke University Institutional Animal Care and Use Committee and were in accordance with NIH Guidelines for the Use of Laboratory Animals. Four adult rhesus macaque monkeys (*Macaca mulatta*) participated in this study. Monkey M and K were chronically implanted with four and six 96 channel arrays, respectively, in bilateral arm and leg areas of M1 and S1 cortex. Monkey C was implanted with eight 96 channel multielectrode arrays in bilateral M1, S1, dorsal premotor (PMd), supplementary motor area (SMA), and posterior parietal cortex (PPC)^36^. In Monkeys M, C, and K, the multielectrode array consisted of a 4 × 8 (M) or 4 × 10 (C,K) grid of cannulae containing microwire bundles which enable different depths of cortical tissue to be sampled. Microelectrode array design and surgical procedure has been previously described 35,36,44. Monkey O was implanted with four 448-channel multielectrode arrays, one each in left and right hemisphere M1 and S1^36^. In each of these arrays, shafts that contained the microwires were arranged in a 10 × 10 layout. Each shaft consisted of 3-4 microwires with 500 μm tip spacing or 6 -7 microwires with 1000 μm tip spacing. For experiments in this study, the recordings in were drawn from M1 (all monkeys) and S1 (only monkey M) cortical areas.

### Experimental Setup

Prior to experiments of this study, each monkey was trained to perform centre-out reaching movements using their left arm to move a spring-loaded joystick. For hand control versions of shared control, both monkeys moved the joystick. For all brain control experiments, the upper limbs of each participating monkey were gently restrained using an established technique^38^. Approximately 50 cm in front of each monkey, at eye level, a computer monitor (26 cm × 30 cm.) displayed the virtual avatar limb^9^,^38^ and target objects from a first-person perspective. The virtual environment was created using Motionbuilder software (Autodesk, Inc.). In all configurations of the task, each participating monkey was placed into a different experiment room with no visual or auditory contact with the other monkeys.

During experiments, neural activity was recorded from each monkey using commercially available Plexon systems (Plexon, Dallas, TX). Spike timestamp information from each of the four (for dyad experiments) or five (for triad experiments) recording systems was sent over the local network to a master computer for neural decoding steps. The master computer contained the BMI software suite, which controlled task flow, decoder input/output, and reward delivery. Once kinematic predictions were generated by the BMI, the avatar movement commands were sent over UDP packets to Motionbuilder in order to update the position of the avatar limb being displayed on the screens. In all dyad experiments, the two monkeys were provided identical visual feedback. In the triad experiment, the visual feedback was different for each monkey.

### Dyad Task Paradigms

#### Shared control task

A circular target (5 cm diameter) appeared in the centre of the screen. The monkeys must hold the avatar arm within the centre target for a random hold time uniformly drawn from the interval 500-1200 ms. Next, the central target disappeared and was replaced by a circular disc (5 cm. in diameter) in one of 4 peripheral locations (left, right, above, or below the centre-point on the screen). The centre of the target disc was 9 cm from the centre. When the monkeys placed the arm within the target such that the cursor (0.5 cm. diameter) at the base of the middle finger (Fig. 1C) was completely within the target, for a hold interval (500 ms), the trial was considered correct and a small juice reward was dispensed. Note that the cursor is shown for illustration purposes; it was not visible to the monkey. Trials where the target was not reached within 10 seconds (hand control) or 15 seconds (brain control) were considered error trials, resulting in no juice and a 1 second timeout period.

The position of the avatar arm was exactly the average of the position derived from each of the two monkeys. In hand control, this was the mean (X,Y) joystick position. For brain control experiments, the neural decoder was fit using 5-6 minutes of data collected during passive observation. The neural decoding algorithm used throughout this study was the unscented Kalman filter (UKF)^37^. During brain control, the avatar hand (X,Y) position was decoded from each monkey’s brain individually, then averaged. Next, the averaged position was used to update the avatar hand position, thus providing the visual feedback to both monkeys.

#### Passive observation

During this task mode, each of the two monkeys in the dyad passively observed identical, pre-programmed reach trajectories on the screen^38^. The hand moved along the ideal trajectories between centre and peripheral target starting ~500 ms after target onset. The observed hand accelerated and decelerated in a realistic manner when starting and stopping a reach. Juice rewards were dispensed when the hand reached and held inside the displayed target. This mode was used for 6-7 minutes at the beginning of all brain control experiments.

#### Partitioned control task

All sessions began with 5-6 minutes of passive observation trials. In this task, all targets appeared at the diagonal locations: 45°, 135°, 225°, and 315° in Cartesian space. Both the monkeys viewed the hand move along the ideal trajectories between centre and peripheral target. Centre and peripheral target sizes, the distance of the target from the centre were the same as in the previous task. Following passive observation, one UKF model was fit for each monkey. The UKF models for monkeys M and C were used to decode only X or only Y, respectively. Next, the task was switched to brain control. Monkey M was given full control of the X position of the avatar hand, and monkey C was given full control of Y position. Together the dyad had to reach toward the target to complete the trial successfully. The pair had to work together to move the hand to the centre, hold briefly, and then move out to the peripheral target, then hold. The hold time and reward contingencies were retained from the previous task. A total of 8 such experiments were completed over a span of three weeks.

#### Triad Task Paradigm

Monkeys M, C, and K formed a Brainet triad to generate 3D reaching movements of the virtual avatar arm. Once again, centre-out movements were performed. Here the peripheral target was located at one of the 8 equidistant corners of a 3D cube. The projected target was 5cm. in diameter and its centre was located 7 cm from the centre of the screen. Each monkey observed only a 2D projection of this movement on their display screen. Monkeys M, C and K were shown the target from an X-Y, Y-Z, or X-Z reference frame respectively. The monkey viewed the avatar arm always from a first-person perspective.

Sessions began with 6-7 minutes of passive observation trials viewed from each monkey’s designated reference frame. Three UKF decoders, one for each monkey, were then initialized and fit on the passive observation data. Each UKF was trained to decode the two dimensions that could be viewed on that monkey’s screen (e.g. X and Y for monkey M). Thus, two UKF models represented X, two represented Y, and two represented Z. Pairs of monkeys with a common dimension were given 50% control of that axis. More specifically, Monkeys M and K each had 50% control of the X axis, Monkeys M and C each had 50% control over the Y axis, and Monkeys C and K each had 50% control of the Z axis. The decoded and averaged X, Y, and Z positions derived from the Brainet triad were then used as a control signal for resultant 3D movements. 2D movement projections were shown as visual feedback to each monkey. For analysis purposes, we considered a monkey to have contributed in a trial if the movements generated by that monkey covered at least 25% of the total distance.

### Analyses

All the analyses were performed in Matlab (Mathworks Inc.) using built-in functions, open-source code, or custom-designed analysis tools developed within the Duke University Center for Neuroengineering.

#### Analysis of neuronal activity

Neurons that were not active for greater than 50% of the session duration were considered quiet neurons and were removed. For the rest, recorded action potential events were counted in bins of 50 ms width and aligned on target onset to obtain PETHs. PETHs were typically calculated for each trial and then were averaged across trials. The average modulation profile for each neuron was normalized by subtracting the mean bin count and dividing by the standard deviation of the cell’s bin count; both values were calculated for raw spike trains prior to any PETH calculations. With this normalization, PETHs express the event-related modulations as a fraction of the overall modulations, or statistically, the *z-*score.

#### Estimating reaction time lag

The joystick positions (in hand control) or the UKF outputs (in brain control) of the two monkeys were cross-correlated after subtracting the data mean and normalizing to a -1 to 1 scale. The position of the peak of the cross-correlation relative to the ordinate provided an estimate of the time lag between the dyad members. Lag was calculated for every rewarded trial and then averaged across all trials to get an estimate of the average lag between the dyad members in a session.

#### Estimating neural activity lag

Temporal relationships between the neural activity of monkey dyads was determined using a cross-correlation with the mean subtracted and normalized to set the auto-covariance terms equal to 1. This allowed the cross-correlations between different trial conditions to be comparable. To compute the between-monkey lag, the single trial PETHs (spike counts within 50 ms bins during a fixed −0.5 to 1 s window relative to target appearance) were collected for each neuron on each trial. All pair wise combinations between Monkey 1 cells and Monkey 2 cells were cross-correlated. This yielded a mean cross-correlogram for each trial, as shown in the left panels in Fig. 2G,H. The time of the peak of the cross-correlogram on each trial was identified and defined to be the single-trial lag between the two monkeys. Mean lag for a session was computed by averaging across trials (Fig. 2F).

#### Trial duration

Trial durations were calculated from the target onset time to when the reward was delivered. To avoid potential confounds due to satiation effects, mean/ average trial duration (Fig. 2C,D, Fig. 4B) was estimated during the initial 15 minutes of every session when the motivation was highest. However, trial durations shown in Fig. 2A,B and Fig. 4A include all trials in the session.

#### k-NN prediction of trial outcome

To predict trial outcome based on level of between-monkey correlation, single trial PETHs were computed for each neuron on each trial. The “pre-target” window was defined as from 700 ms before target appearance until target appearance. The “post-target” window was defined as from 100 to 800 ms after target appearance. The activity profile of each neuron during the specified epoch was normalized and then cross-correlated with the activity profile from each neuron from the other monkey. The cross correlogram for each pairwise neuronal combination was then averaged to produce a single between-monkey cross correlogram for a given trial. The set of single-trial cross correlograms were then used to predict the outcome of their corresponding trials or that of a randomly chosen trial (control). 80% of trials were designated to be training data and 20% of trials were test data. A k-nearest neighbour classifying algorithm (k-NN with k = 5) was fit and then tested by predicting trial outcome on the designated test trials. Performance was quantified in terms of fraction correct prediction. Training/test data was redrawn 10 times. We reported the distribution of the fraction correct values after redrawing the training/test data (Fig. 2I,J).

#### Neuron dropping analysis

Neural activity was recorded and binned into 100 ms bins during passive observation experiments. An unscented Kalman filter (UKF) with three 100 ms past taps of neural activity and two 100 ms future taps of neural activity was used to decode the X and Y position of the observed avatar hand on the screen. UKF prediction of X and Y were computed for one of the monkeys for a wide range of neurons, ranging from 1 to the full ensemble, in increments of 3 (n <  20), 7 (at 20 < n < 100), or 15 (n > 100). The predicted X and Y traces were compared with the actual X and Y traces, the correlation coefficient *r* was computed for each, and then averaged. In B2 configurations, a second monkey was then added to the analysis. X and Y UKF predictions using 25%, 50%, 75% and 100% of the 2^nd^ monkey’s neurons were then computed. The predicted X and Y were averaged with the X and Y from Monkey 1’s prediction, as was done during on-line experiments. The accuracy of these averaged predictions was quantified using correlation coefficient as well.

In B3 configurations, the predictions were generated in a similar way as for B2. The two coordinates controlled by each monkey (X-Y, Y-Z, or X-Z) were likewise decoded from the ensembles of varying sizes using a UKF decoder. For X, Y, and Z, predictions were averaged across the two monkeys who shared each axis. For example, the X predictions from Monkey M and K were computed and averaged to produce one *r* value for each of the different quantities of Monkey M and K neurons. Fixed percent quantities of the second monkey were added to the first monkey’s ensemble to show the effect of adding neurons from different subjects in a Brainet.

#### Normalized contribution

In a trial, the fraction of the total distance moved by each monkey in the B3 was computed along each axis. A monkey’s normalized contribution (Fig. 4C) was calculated by summing its contributions (in the two directions) and normalizing it by dividing by 3.

Negative values (capped at -0.2) indicate movements generated in the direction away from the target.

#### ANOVA on bootstrapped samples

All the trials from a session were resampled several times with replacement and the fraction of error trials, rewarded trials contributed by all the members of the triad, or by a dyad was computed for each sample. A one-way ANOVA was performed on these samples across sessions. F value of the original sample was compared with those of the bootstrapped samples.

## Additional Information

**How to cite this article**: Ramakrishnan, A. *et al.* Computing Arm Movements with a Monkey Brainet. *Sci. Rep.* **5**, 10767; doi: 10.1038/srep10767 (2015).

## Supplementary Material

Supplementary Information

Supplementary Movie 1

Supplementary Movie 2

Supplementary Movie 3

## Figures and Tables

**Table 1:** 

Group name	Acquisition time	Precision	Hit rate
HC Dyad MO	0.7s	2.04	82.02%
B2: Dyad MO	1.86s	2.04	86%
B2: Dyad MC	1.75s	2.04	80%
B3: Triad MCK	3.31s	0.37	78%
