# Supplementary material for: Computing Arm Movements with a Monkey Brainet
Source: Sci Rep. 2015 Jul 9;5:10767. doi: 10.1038/srep10767 (PMC4497496; doi:10.1038/srep10767)
Supplement: Supplementary Information [file srep10767-s1.doc]

**Supplementary Information**

**Computing Arm Movements with a Monkey Brainet**

Arjun Ramakrishnan, Peter J. Ifft, Miguel Pais-Vieira, Yoon Woo Byun, Katie Z. Zhuang, Mikhail A. Lebedev, Miguel A.L. Nicolelis

**Supplementary Figures and Legends**


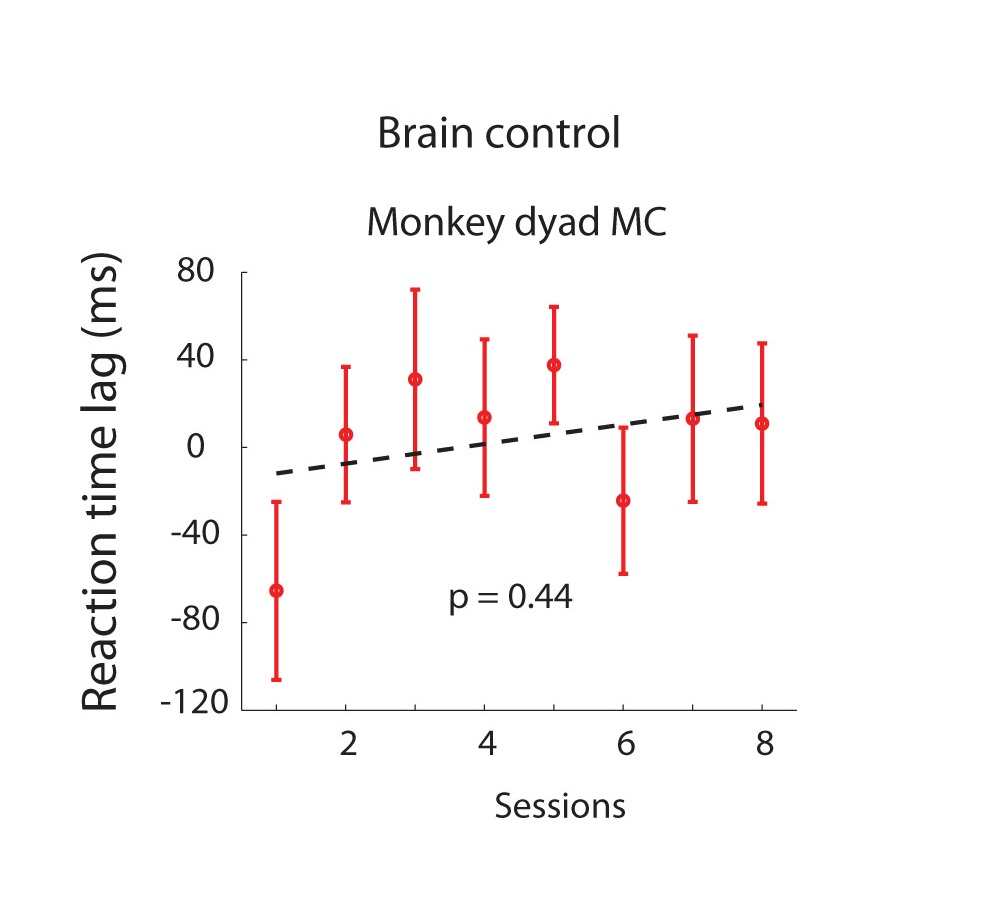


**Figure S1:** Changes in reaction time lag between monkeys over training for dyad M&C. Reaction time lag was derived from peak of cross-correlation of two monkeys’ behavioural traces (see Fig. 2E-F). Trends were fit with linear regression. The behavioural responses were highly synchronous, with lag remaining close to zero (3±35ms) throughout all sessions.

**
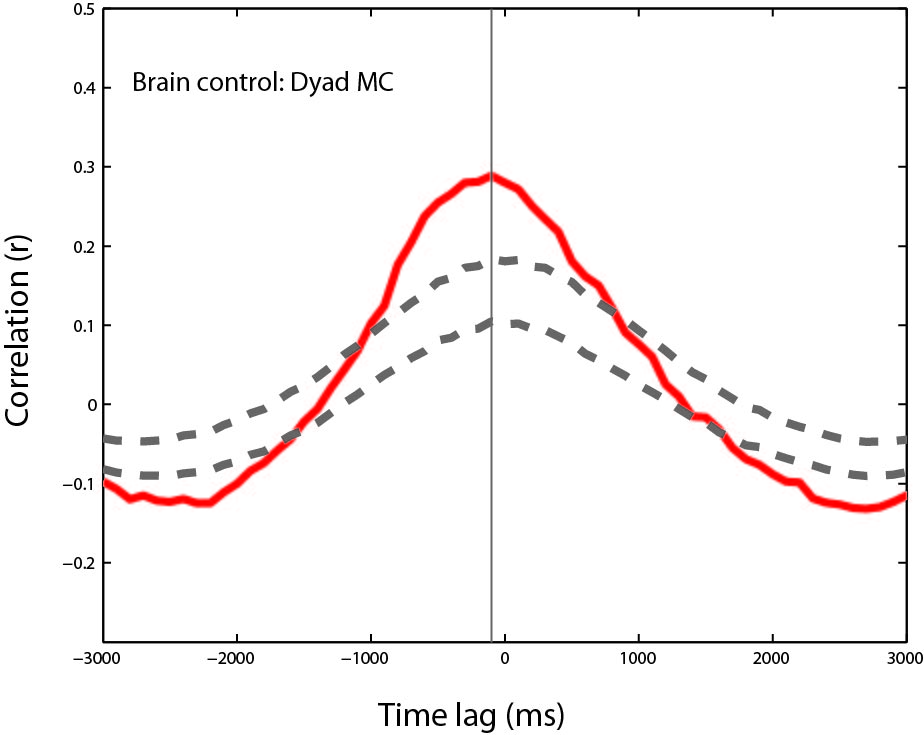
**

**Figure S2:** Extra correlation analysis for dyad M&C. Velocity profile in a trial for dyad M&C were cross-correlated. The average cross correlation (trial specific correlation, red trace) was estimated for all trials. Extra correlation is the excess correlation (in the red trace) that cannot be accounted for by the distribution of across-trial correlation (grey distribution). The vertical line shows the time lag at peak correlation.

**Supplementary Movie Legends**

**Movie S1:** Monkey Dyad M&O contributions during shared control trials. Shown separately are Monkey O’s contribution (red dot), Monkey M’s contribution (blue dot), and the average (black dot) from a late session. The average of the two was used to control the avatar, which was used for visual feedback. Notice the synchronized movements by the dyad.

**Movie S2:** Monkey Dyad M&C contributions during shared control trials. Shown separately are Monkey C’s contribution (red dot), Monkey M’s contribution (blue dot), and the average (black dot) from a late session. The average of the two was used to control the avatar, which was used for visual feedback. The dyad movements are synchronized as in S1.

**Movie S3:** Monkey triad M, C and K contribute during the 3D task. Shown separately are Monkey C’s contribution (red dot), Monkey M’s contribution (blue dot), and Monkey K’s contribution (green dot) and the average (black dot) from a late session. Note that each monkey controls only 2 of the 3 axes.
